# Bilateral Posterior Fracture-Dislocation of the Shoulders Secondary to Uremic Encephalopathy

**DOI:** 10.5435/JAAOSGlobal-D-20-00255

**Published:** 2023-01-05

**Authors:** Marlon M. Mencia, Raakesh Goalan

**Affiliations:** Department of Clinical Surgical Sciences, University of the West Indies, Port of Spain, Trinidad and Tobago (Mencia), and Department of Orthopaedics, Eric Williams Medical Sciences Complex, Trinidad and Tobago (Goalan).

## Abstract

Bilateral posterior fracture-dislocation of the shoulder is an uncommon injury pattern usually caused by epileptic seizures. The cause of the seizure activity remains unknown in most cases, although the injury has been associated with several conditions. A 59-year-old man with uncontrolled hypertension presented with new-onset generalized tonic-clonic seizures. He was diagnosed with uremic encephalopathy and bilateral posterior fracture-dislocation of his shoulders. His medical condition required stabilization leading to a delay in definitive surgery and a subsequent poor outcome. This case highlights the previously unknown association between bilateral fracture-dislocation of the shoulders and seizures caused by uremic encephalopathy. In these complex situations with competing clinical priorities, it is important to initiate prompt treatment of the cause in any new-onset seizures, to facilitate expedient surgical management of the orthopaedic injury.

The shoulder is a ball and socket synovial joint and is easily the most commonly dislocated joint in the body. Although dislocation can occur in several directions, posterior dislocations are relatively rare and comprise only 1.7% to 4.3% of all cases, of which less than 1% is associated with a fracture.^1,2,3,4,5,6^ Studies show that 5% of all posterior fracture-dislocations occur bilaterally; it follows, therefore, that few physicians will ever encounter this scenario in the clinical setting.^7,8^

The common etiologies for posterior fracture-dislocation of the shoulder have been described by Brackstone et al who proposed the “triple E syndrome”: epileptic seizures, extreme trauma, or electrocution, with bilateral injuries being almost pathognomonic of an epileptic etiology.^6,9,10,11,12,13,14,15,16,17^ The commonest cause of a seizure leading to a bilateral posterior fracture-dislocation of the shoulder is idiopathic epilepsy; although other causes have been described, there are no reports of its association with uremic encephalopathy (UE).^3,9,12,18-20^

We present a case of bilateral posterior fracture-dislocation of the shoulder secondary to a seizure caused by UE. To the best of our knowledge, this injury pattern in a patient with undiagnosed chronic kidney disease (CKD) has not been previously described in the literature.

## Case Report

A 59-year-old man with uncontrolled hypertension presented to the emergency department with new-onset generalized tonic-clonic seizures. A family member who witnessed the episode confirmed that the patient was noncompliant with his antihypertensive medication and over the preceding months exhibited signs of memory loss and depression.

On admission, his initial blood pressure recording was 210/100 mmHg, and the complete blood count showed a severe normocytic, normochromic anemia with a hemoglobin level of 5.6 g/dL. Renal function tests showed a blood urea nitrogen of 145 mg/dL, creatinine (Cr) of 17 mg/dL, estimated glomerular filtration rate 3 mL/min with potassium (K) of 6.6 mg/dL, taken all together this suggested long-standing severe CKD.

**Figure 1:**
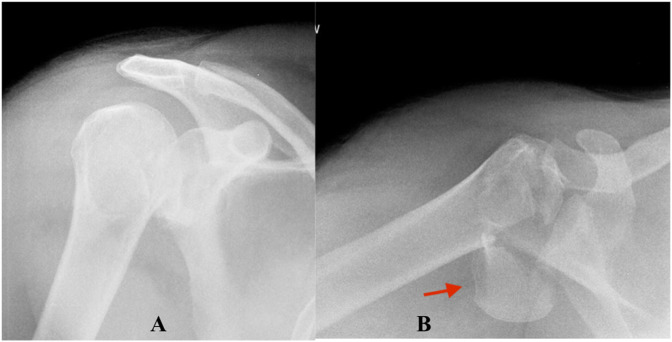
Anteroposterior (**A**) and lateral (**B**) radiographs demonstrating a posterior fracture-dislocation of the right shoulder (note red arrow showing a large humeral head fragment).

**Figure 2:**
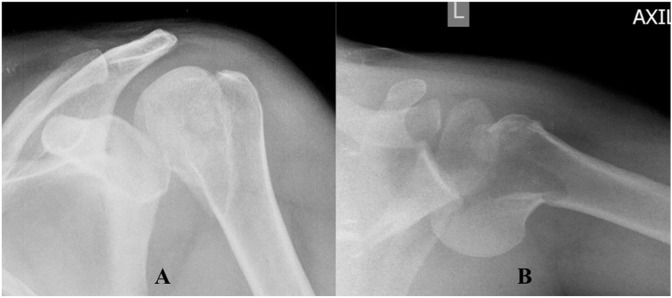
Anteroposterior (**A**) and lateral (**B**) radiographs demonstrating a posterior fracture-dislocation of the left shoulder.

The patient's seizures were brought under control, and in the post-ictal state, he began to complain of pain in both shoulders. The on-call orthopaedic resident was summoned and in his examination noted loss of the normal shoulder contour bilaterally. In addition, the patient's upper limbs were found to be slightly internally rotated and attempted active or passive shoulder movements were painful and accompanied by crepitus. Emergency department radiographs demonstrated bilateral posterior fracture-dislocation of the shoulders (Figures 1 and 2).

**Figure 3:**
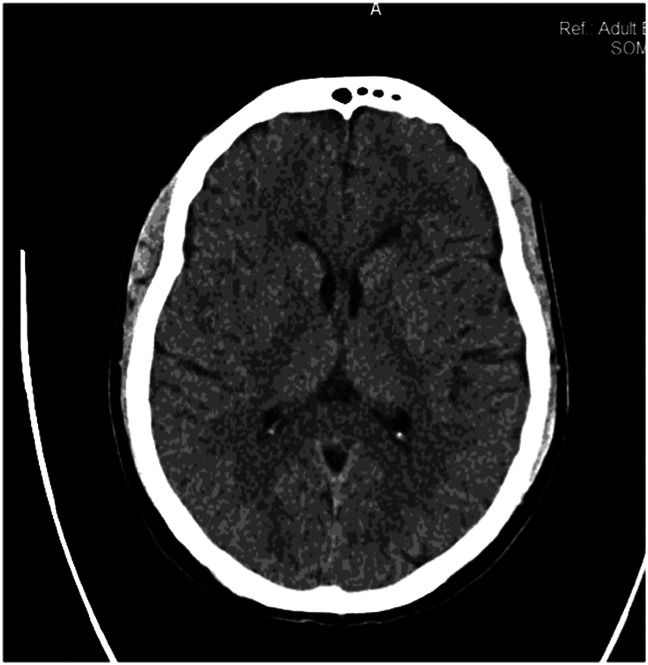
Axial noncontrast CT of the brain showing no abnormalities. CT = computed tomography

**Figure 4:**
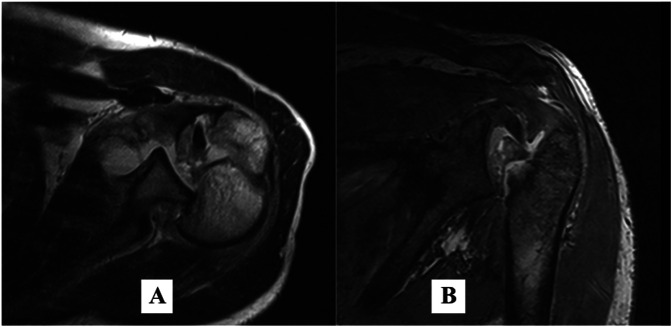
T2-axial (**A**) and T1-coronal (**B**) MRI of the left shoulder joint showing a displaced posterior fracture-dislocation.

Computed tomography (CT) scan of his brain revealed no space-occupying lesions, which led his physicians to believe that the seizures were secondary to UE Figure 3. He underwent urgent hemodialysis to correct the metabolic and electrolyte imbalances, in addition to a blood transfusion to treat his anemia. Over the next week, as his blood pressure returned to normal and his biochemical parameters stabilized, there was considerable clinical improvement with no apparent cognitive dysfunction. During this period, we requested a preoperative surgical planning CT scan of both shoulders; unfortunately, the CT scanner was inoperable, and the patient was referred for MRI Figure 4. The patient continued to recover slowly, and 4 weeks after admission, he was medically cleared to proceed with surgery.

The patient was taken to the operating theater and under general anesthesia placed in the beach-chair position. First, the right side was approached by way of a deltopectoral incision. The bone quality was found to be adequate, and using fluoroscopic guidance, the shoulder was reduced, and the fracture stabilized with a four-hole proximal locking the humeral plate. After soft-tissue closure, the joint was cycled through a functional range of movement and was found to be stable. Attention was then directed to his left shoulder, which was approached similarly. After surgical exposure, abundant callus was noted at the fracture site, and the overall position was thought to be acceptable. Having decided not to take down the fracture, we reduced the joint, but excessive soft-tissue scarring rendered it unstable during passive movement. With the humeral head reduced, a single percutaneous 2.0-mm K-wire placed across the shoulder joint was used to maintain the reduction. The soft tissues were then meticulously closed, and his arm was supported with a shoulder immobilizer. All incisions healed *per primam.*

**Figure 5:**
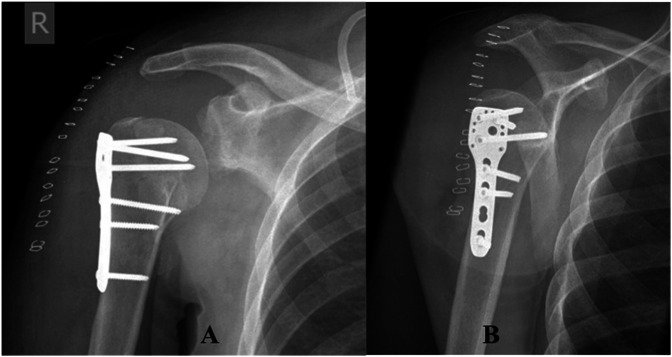
Anteroposterior (**A**) and lateral (**B**) radiographs showing a good reduction of the right shoulder using a 4-hole humeral locking plate

**Figure 6:**
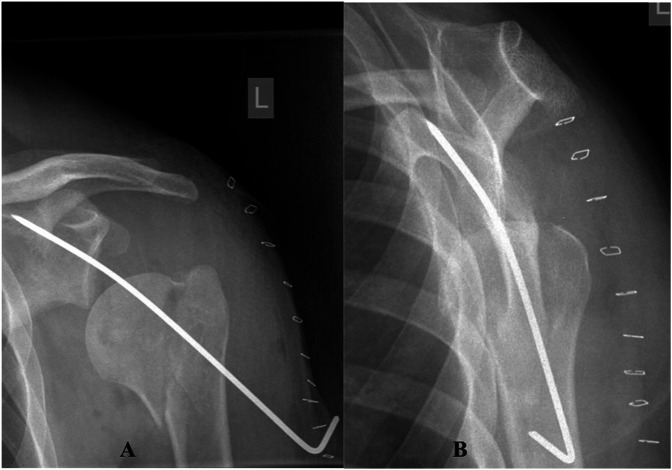
Anteroposterior (**A**) and lateral (**B**) radiographs of the left shoulder illustrating a satisfactory reduction of the fracture-dislocation but with significant inferior subluxation of the humeral head.

Postoperative radiographs were satisfactory on the right with a small degree of inferior subluxation caused by deltoid inhibition. The left side showed significant inferior subluxation from the weight of his upper limb, but the humeral head remained reduced on the lateral view (Figures 5 and 6). Early physical therapy was started on the right and 4 weeks later on the left side after removal of the Kirschner wire.

**Figure 7:**
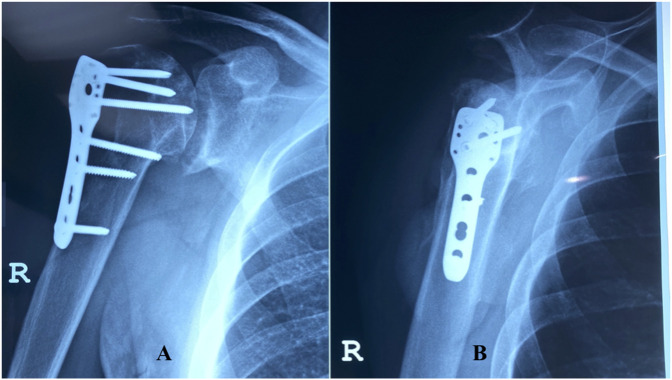
Anteroposterior (**A**) and lateral (**B**) radiographs of the right shoulder showing early degenerative changes at the glenohumeral joint.

**Figure 8:**
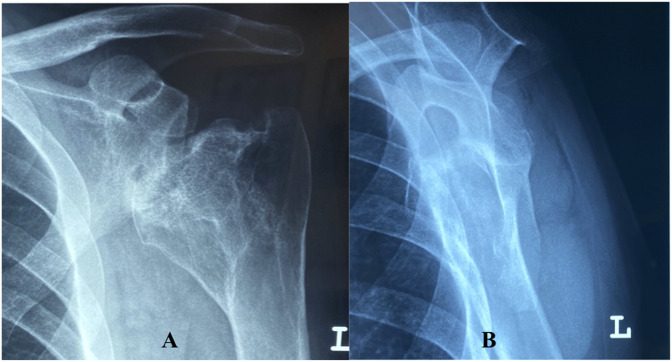
Anteroposterior (**A**) and lateral (**B**) radiographs of the left shoulder illustrating humeral head collapse with subluxation and advanced degeneration at the glenohumeral joint.

**Figure 9:**
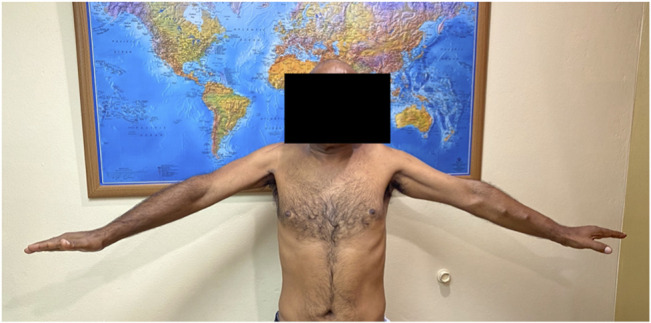
Clinical photograph demonstrating limited abduction

**Figure 10:**
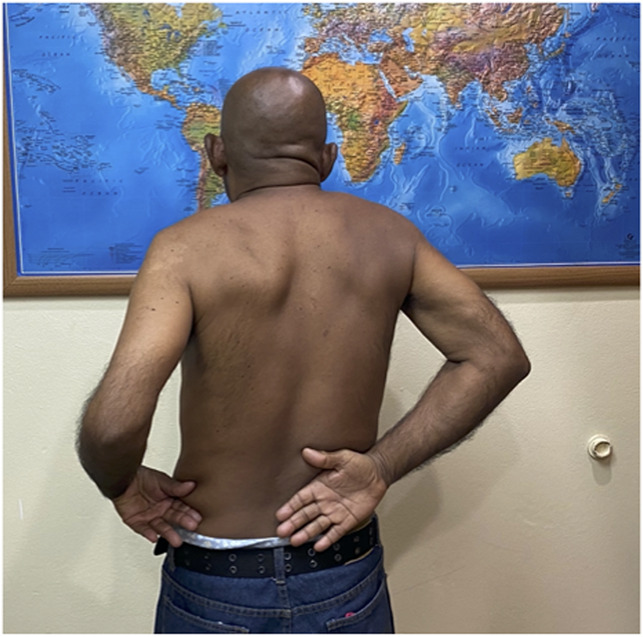
Clinical photograph demonstrating limited internal rotation, more obvious on the left

**Figure 11:**
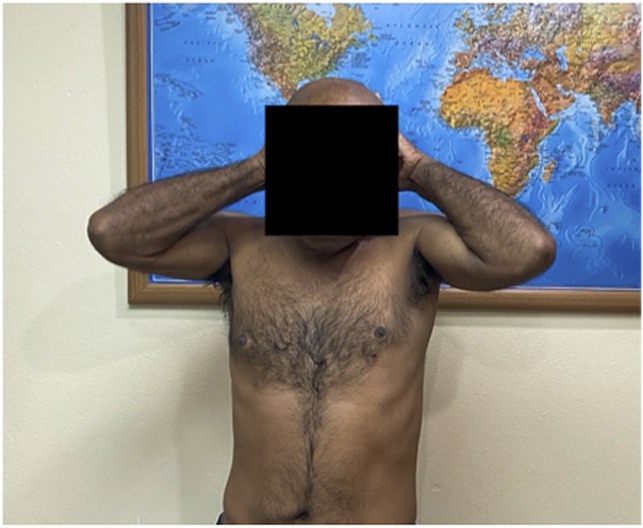
Clinical photograph demonstrating limited abduction and external rotation

The patient was followed up regularly in the clinic with annual radiographs. (Figures 7 and 8). Three years after his injury, he reports only mild shoulder discomfort with limited movement, particularly on the left and is now able to work part time while having twice weekly hemodialysis (Figures 9–11). His Oxford Shoulder Score, Disabilities of the Arm, Shoulder and Hand Score (QuickDash) and EQ-5D are shown in Table 1.

**Table 1:** Patient Reported Outcome and Health Related Quality of Life Assessment

	Right	Left
Oxford Shoulder Score	41	34
QuickDash Score	18.2	31.8
EQ-5D	0.864	

The patient gave informed consent for his case data to be used in this report.

## Discussion

Bilateral posterior fracture-dislocation of the shoulder is thought to be pathognomonic of an epileptic seizure.^6,9,10^ The injury pattern is associated with several conditions; however, no aetiological cause is ever identified in most cases.^18,21^ Noncompliance with antihypertensive medication for several years was the likely reason for our patient developing CKD. His preseizure deterioration in cognitive ability, together with his metabolic and electrolyte abnormalities on admission, suggested a metabolic derangement with UE most often being the cause of seizures.^22^ The increased permeability of the blood-brain barrier in CKD leads to a higher concentration of guanidino compounds in the cerebrospinal fluid, resulting in heightened cortical excitability, thereby lowering the seizure threshold.^23^ UE was recognized early in our patient, and prompt treatment was initiated, including urgent hemodialysis and blood transfusion. The patient responded well to treatment, with no further seizures has remained on long-term hemodialysis.

Bilateral posterior fracture-dislocations of the shoulder are commonly missed on initial presentation, leading to a delay in treatment and inferior clinical outcomes.^6,24^ The key to early diagnosis of these rare injuries is maintaining a high index of clinical suspicion and requesting appropriate imaging. Of the three views recommended for acute shoulder trauma (trauma series), the lateral view is of critical importance in identifying a shoulder dislocation. In a systematic review of posterior dislocation of the shoulder, the authors report an initial misdiagnosis rate of 73.2% (150/205); of these patients, almost all (98%, 147/150) had only an anterior-posterior radiograph. The diagnosis was later confirmed in all 205 patients after axillary or scapular Y lateral view radiographs.^25^ The axillary lateral view, which requires approximately 70° to 90° of abduction, is not always possible in acute trauma. In these cases, the Velpeau view permits an axillary lateral radiograph with the patients' arm comfortably stabilized in a sling.^26^ When a shoulder dislocation is missed and surgery is delayed for more than three weeks, closed treatment is unlikely to succeed.^27^

Notwithstanding the prompt diagnosis of our patient's orthopaedic injury, definitive surgical management was postponed because treatment of his CKD required more urgent coordinated management involving other specialties. Inconsistent hemodialysis caused by institutional shortcomings resulted in a delay of 4 weeks before surgery. At surgery, we encountered significant fibrous tissue, chondrolysis and malunion, particularly on the left side, which we believe contributed to an inferior clinical result.

The use of MRI, in this case, is controversial. Cross-sectional imaging, specifically CT, is typically recommended before surgery.^28^ Voight et al evaluated the diagnostic values of conventional radiographs, CT, and MRI for proximal humeral fractures, concluding that although radiographs were sufficient for the diagnosis, both CT and MRI were equally good at demonstrating complete fracture patterns, with MRI giving additional information about the rotator cuff and head perfusion.^29^ We encountered no difficulty interpreting the MR scans and found the T1-weighted coronal and axial images to be the most useful for preoperative planning, but considering the increased cost, cannot recommend its routine use.

There is no consensus on the most appropriate surgical management for posterior fracture-dislocations of the shoulder. The classification system proposed by Robinson et al^30^ attempts to rationalize treatment options.^31^ Hertel et al^31^ have described radiological features, which they believe are predictors of humeral head ischemia as the main drivers of treatment. In practice, surgical decision-making is a complex process dependent on several factors including surgical expertise, instrumentation and implants, bone quality, and the general condition of the patient. We treated both shoulders differently based on the findings at surgery. Local conditions, including excessive scar tissue, caused by the delay in getting to surgery, prevented conventional treatment of the left side.

Our management of the left shoulder may be considered unconventional and deserves further explanation. At surgery, we found the fracture to be in an advanced stage of healing. Taking the fracture down and then using a locking plate for stabilization would have required elaborate surgery with the risks of neurovascular damage, loss of vascularity to the humeral head, and deep infection. Under the circumstances, we felt that the benefits of this approach were limited and, therefore, did not consider it further. The use of pins or Kirschner wires in the shoulder region is associated with a propensity for migration and breakage, resulting in significant mobility and mortality. Lyons and Rockwood, in their classic review of wire migration in the shoulder, recommended utmost caution when using wires, citing the risk of catastrophic cardiovascular complications and death.^32^ We used one smooth 2-mm Kirschner wire to stabilize the shoulder joint, which readily bent under the weight of the patient's arm. In retrospect, it would have been better to use at least two heavy Kirschner wires (>3 mm), which may have prevented the inferior subluxation seen in the early postoperative period.^32,33^

Inferior subluxation of the shoulder joint was noted soon after surgery and was present at the final follow-up. Transient postoperative inferior subluxation may be caused by deltoid atony or the loss of negative intra-articular pressure. Pritchett reported an incidence of 42% for radiographic inferior subluxation after fracture of the proximal humerus, with 92% of cases resolving by six weeks and complete resolution at 2 years. The author recommended the use of a sling and early active exercises as effective treatment measures.^34^ We postulate that inadequate initial reduction and limited rehabilitation contributed to the lasting inferior subluxation. At one year, radiographs show early post-traumatic glenohumeral arthritis, and comparative analysis demonstrates inferior function on the left compared with the right shoulder.

Bilateral posterior fracture-dislocation of the shoulder is a rare occurrence, commonly associated with epileptic seizures and frequently missed on initial presentation. A delay in definitive treatment can lead to inferior clinical outcomes. This case report highlights the importance of identifying the cause of the seizures and initiating appropriate treatment to facilitate timely surgical intervention. High endemic rates of diabetes mellitus and hypertension, together with poor compliance and drug shortages, have resulted in increasing rates of CKD in the Caribbean.^35,36^ CKD is a rapidly growing global health problem, and surgeons should be aware that UE may cause “unexplained” seizures.^37^ To the best of our knowledge, this is the first case that links bilateral posterior fracture-dislocation of the shoulder with undiagnosed CKD and UE.
